# Multidisciplinary Management of Paediatric Nasolacrimal Duct Obstruction at a Tertiary Hospital: A Five-Year Review

**DOI:** 10.7759/cureus.106846

**Published:** 2026-04-11

**Authors:** Arshad Zubair, Ivan Yip, Jose Gonzalez-Martin, Ravi Sharma, Grace Khong

**Affiliations:** 1 Otolaryngology - Head and Neck Surgery, Alder Hey Children's NHS Foundation Trust, Liverpool, GBR; 2 Paediatric Ophthalmology, Alder Hey Children's NHS Foundation Trust, Liverpool, GBR; 3 Otolaryngology, Alder Hey Children's NHS Foundation Trust, Liverpool, GBR

**Keywords:** congenital nasolacrimal duct obstruction, craniofacial abnormalities, dacryocystorhinostomy, down's syndrome, nasolacrimal duct system

## Abstract

Introduction

Nasolacrimal duct obstruction (NLDO) is the most common cause of childhood epiphora. Congenital NLDO is usually managed conservatively in the first year of life; failing which, surgical interventions - such as syringing and probing (S&P), insertion of stents (intubation), or dacryocystorhinostomy (DCR) - are offered in a stepwise manner. The aim of this study was to evaluate the outcomes of our joint ophthalmology-ENT service in the management of NLDO.

Methods

This was a retrospective study conducted at a tertiary paediatric hospital. Nasolacrimal surgeries were retrieved from Hospital Episode Statistics (HES) data for a five-year period between May 2017 and April 2022. A retrospective case-note review was undertaken to examine demographics, presentation, surgical interventions, and outcomes (classified as complete resolution, partial improvement, or no improvement).

Results

At our institution, NLDO surgeries are performed on a joint ophthalmology/ENT list. A total of 301 procedures were performed on 218 patients (293 eyes). The causes of epiphora included congenital NLDO (n = 193, 88.5%), secondary NLDO (n = 10, 4.6%), and dacryocystitis/mucocele (n = 8, 3.7%), among others. The median age at the first procedure was 26 months (range: 2-189). The median number of procedures for congenital NLDO was one (range: 1-5). The success rates were 73% for all S&P procedures (n = 135/185), 78% for intubation (n = 76/98), and 58% for DCR (n = 10/17). Patients with craniofacial syndromes required a statistically significantly higher number of DCRs. Overall, epiphora was completely resolved in 81% of cases (n = 237), partially resolved in 6% (n = 18), and was persistent in 13% (n = 38).

Conclusion

A multidisciplinary approach to NLDO ensures the efficient delivery of care by minimising the number of procedures and hospital attendances. Congenital NLDO can be successfully treated in the vast majority of cases with S&P and intubation. In cases with bony anatomical abnormalities, there should be a low threshold for considering DCR.

## Introduction

Nasolacrimal duct obstruction (NLDO) is a common condition in the paediatric population. It is most often congenital NLDO (cNLDO), with a reported incidence of 11%-20% in newborns [1]. This obstruction results from the incomplete canalisation of the nasolacrimal duct (NLD) during embryonic development. While the most common site of obstruction in cNLDO is at the distal end of the NLD, at the membrane of Hasner, it can also arise from bony abnormalities or more proximal obstructions involving the canaliculi and lacrimal sac. Secondary (acquired) causes of paediatric NLDO include post-traumatic obstruction of the lacrimal system, infections (such as dacryocystitis), and inflammatory or neoplastic conditions.

A conservative approach is widely recommended as the first-line management for up to 12-18 months of age, as the rate of spontaneous resolution for cNLDO by the end of the first year is as high as 96% [2]. This approach includes the use of topical ocular antibiotics in addition to regular lacrimal sac massage. Persistent symptoms may warrant surgical intervention, which is offered in a stepwise manner: syringing and probing (S&P), intubation, and dacryocystorhinostomy (DCR). There is significant variability in the choice of surgical interventions across the UK, particularly in the use of a nasal endoscope. According to a recent survey of paediatric ophthalmologists and oculoplastic surgeons in the UK, only 27% of primary S&P procedures are performed under nasal endoscopic guidance [3]. There is growing evidence to suggest that endoscopic-assisted probing improves resolution rates in children compared to conventional (blind) probing [4]. The aim of this study was to evaluate the outcomes of our joint ophthalmology-ENT service in the management of NLDO.

This article was previously posted on the Authorea preprint server on March 14, 2023 [5].

## Materials and methods

This is a retrospective cohort study conducted at a tertiary paediatric centre in the UK. All nasolacrimal surgeries performed at our institution between May 1, 2017, and April 30, 2022, were included. Data were retrieved using Hospital Episode Statistics (HES) (procedural codes C27.1-C27.9). There were no strict exclusion criteria.

Our clinical service for patients referred with a watery eye begins with an evaluation in the paediatric ophthalmology clinic. A diagnosis of NLDO is confirmed with the fluorescein dye disappearance test (FDDT). If the clinical history is suggestive of NLDO, with a high tear meniscus on examination, the FDDT is not undertaken. Patients with cNLDO are managed conservatively for up to 12-18 months of age and are primarily advised to perform lacrimal massage, with occasional use of antibiotic eye drops if there are signs of infection. If symptoms persist or are complicated by recurrent conjunctivitis or dacryocystitis, surgical intervention is offered.

All primary procedures are performed under general anaesthesia, with nasal endoscopic guidance, in a joint ophthalmology-ENT operating list. The majority of patients are consented for S&P as the first intervention unless more extensive interventions, such as intubation or DCR, are anticipated from the clinical history. Indications for probing include persistent epiphora with no spontaneous resolution by the age of 12-18 months, a mucocele of the lacrimal sac, or chronic dacryocystitis or conjunctivitis due to lacrimal sac obstruction.

The patients’ nasal cavities are prepared with adrenaline-soaked neuropatties, which are placed in the inferior meatus before surgery. A Bowman’s probe is passed through the superior or inferior punctum. The inferior meatus is visualised using a zero-degree endoscope (Storz, Tuttlingen, Germany), and true probe passage through the valve of Hasner is confirmed. Common intranasal interventions performed along with S&P include excision of a membranous stenosis at the valve of Hasner, in-fracture of the inferior turbinate, and removal of adhesions in the inferior meatus (more commonly in secondary NLDO).

Indications for intubation include the recurrence of symptoms after probing or difficult probing, with a clinical suspicion of early re-stenosis. Intubation is conducted utilising a Masterka monocanalicular silicone stent (Masterka, France Chirurgie Instrumentation SAS, France). Masterka stents are removed three to six months postoperatively during a clinic visit under topical anaesthesia. Rarely, the removal of stents may require general anaesthesia.

DCR is indicated in cases of symptom recurrence after intubation, failed intubation, or in the presence of bony anomalies such as a narrow NLD. Endonasal DCR is performed jointly with the placement of a Crawford’s bicanalicular stent, which is removed three to six months postoperatively in the clinic. The steps of endonasal DCR are as follows: a posteriorly based mucosal flap is raised over the lacrimal bone, which is removed with a combination of a Kerrison rongeur and a drill. The lacrimal sac is opened through a vertical incision. Crawford’s bicanalicular stents are pulled through and secured with a sleeve. The mucosal flap is then repositioned.

Outcomes were classified as complete resolution, partial improvement, and no improvement. The success rate was measured by including both complete resolution and partial improvement, as neither outcome required further surgical intervention. The resolution of symptoms was based on the absence of tearing and discharge, as per clinical assessment and parental history. Partial resolution was defined as a reduction in tearing that did not warrant further surgical intervention.

Approval was received from the Institutional Clinical Audit Team (reference number 6895). Data analysis was performed using RStudio Version 1.3.1093 (© 2009-2022 RStudio, Inc., Boston, MA, USA). Fisher’s exact test was used to examine the association between categorical data.

## Results

A total of 218 patients (293 eyes) with NLDO required surgical intervention. The diagnoses included cNLDO (n = 261, 89%), secondary NLDO (n = 15, 5%), and dacryocystitis/mucocele (n = 12, 4%), among others. The median age at the first intervention was 26 months (range: 2-189 months).

Interventions

A total of 301 interventions were performed on 293 eyes, including 185 S&P (63%), 98 intubations (31%), and 17 DCRs (6%). The median ages at the first S&P, first intubation, and first DCR were 23 months (mean: 31.8; range: 2-177), 45 months (mean: 61.6; range: 15-189), and 101 months (mean: 99; range: 29-189), respectively. The mean follow-up period was 22.5 ± 17.3 months, with a 10% loss to follow-up rate (primarily owing to the COVID pandemic).

The median number of procedures per patient was 1 (range: 1-5). Of the 218 patients, 160 (73.3%) required only a single intervention. These included S&P in 81.1% of cases (n = 130), intubation in 14% (n = 22), and one DCR (n = 1). DCR was performed as the first intervention in a seven-year-old with persistent epiphora following multiple facial fractures sustained after a fall. Due to a failure to intubate the nasolacrimal system, a decision was made to perform an endonasal DCR with a Crawford’s bicanalicular stent. This patient had a complete resolution of symptoms, with no further interventions required.

Outcomes

Complete resolution was achieved in 81% of our patient cohort (n = 237), with partial improvement in 13% (n = 38) and no improvement in 6% (n = 18). The success rates were 73% for all S&P procedures (n = 135), 78% for intubation (n = 76), and 58% for DCR (n = 10).

The reasons for DCR failure in our series included abnormal intranasal anatomy and a narrow lacrimal sac (n = 4), absence of the lacrimal sac (n = 1, in a case of Tessier cleft, which was subsequently managed with a dacryocystectomy via an external approach), and granulation tissue around the Crawford’s tube (which required re-intubation). In two cases, there was a recurrence of symptoms after the removal of the tubes, which was considered to be due to scarring.

Craniofacial (CF) syndromes

There were 15 patients with CF syndromes (Table 1). This cohort required a median of 2 procedures (range: 1-5), with an overall success rate of 66.6% (n = 10). DCR was required in 33% of the CF group (n = 5) compared to 4% of the non-CF group (n = 8) (OR = 10.5, 95% CI = 2.3-43.8, p = 0.001). Our success rate with DCR for the CF group was 25% (n = 2/8), compared to 77.7% (n = 7/9) in the non-CF group (OR = 0.11, 95% CI = 0.005-1.26, p = 0.056).

**Table 1:** Outcomes in craniofacial disorders (n = 15) *Oculo-auriculo-vertebral spectrum DCR: dacryocystorhinostomy; S&P: syringing and probing

No.	Craniofacial disorder	Involved side	No. of procedures	Last intervention	Outcome
1	OAVS*	Bilateral	5	DCR	Complete resolution
2	Tessier cleft	Bilateral	2	Dacryocystectomy	Partial improvement
3	Kabuki syndrome	Bilateral	1	Intubation	Complete resolution
4	18q deletion	Bilateral	1	Intubation	Complete resolution
5	Branchio-oto-renal syndrome	Right	4	DCR	Complete resolution
6	Branchio-oto-renal syndrome	Bilateral	3	Intubation	No improvement
7	Noonan syndrome	Bilateral	1	S&P	Complete resolution
8	Goldenhar syndrome	Left	1	S&P	Partial improvement
9	Alagille syndrome	Right	3	Intubation	Complete resolution
10	Tessier cleft	Right	2	DCR	No improvement
11	Cleft lip and palate	Right	2	S&P	Complete resolution
12	Saethre-Chotzen syndrome	Bilateral	1	S&P	Complete resolution
13	Rothmund-Thompson syndrome	Left	3	DCR	No improvement
14	Tessier cleft	Right	1	S&P	Complete resolution
15	Menke-Hennekam syndrome	Bilateral	1	Intubation	Complete resolution

Down syndrome

There were 12 patients with Down syndrome (Table 2). This cohort required a median of 2 procedures (range: 1-3), with a success rate of 50% (n = 6). Bilateral intubation was required in five patients. Two patients in this cohort are awaiting DCR.

**Table 2:** Outcomes in patients with Down syndrome (n = 12) S&P: syringing and probing

No.	Involved side	No. of procedures	Last intervention	Outcome
1	Bilateral	3	Intubation	Complete resolution
2	Left	2	Intubation	No improvement
3	Right	1	Intubation	No improvement
4	Bilateral	2	Intubation	Complete resolution
5	Left	2	Intubation	No improvement
6	Left	2	Intubation	Complete resolution
7	Bilateral	2	Intubation	No improvement
8	Right	2	S&P	Complete resolution
9	Bilateral	2	Intubation	No improvement
10	Bilateral	2	Intubation	Complete resolution
11	Left	1	S&P	Complete resolution
12	Bilateral	1	S&P	No improvement

## Discussion

This study reports the outcomes of our joint ophthalmology-ENT service in the management of paediatric NLDO. Our service offers nasal endoscopic-guided probing, intubation, and DCR as surgical interventions for pediatric NLDO. A complete resolution of symptoms was achieved in 81% of 293 eyes, with a median of one procedure required per patient.

Joint ophthalmology-ENT service

Outpatient clinic S&P continues to be highly uncommon in the UK, with the vast majority of procedures performed under general anaesthesia [3]. Most paediatric ophthalmologists in the UK consider performing at least two S&P procedures before proceeding with intubation.

Golash et al. reported only a 27% use of the nasal endoscope in primary S&P in a UK survey of paediatric ophthalmologists and oculoplastic surgeons [3]. The use of the nasal endoscope was 25% in a similar study from 2006 [6]. The authors of this survey concluded that this reflects plateaued levels of endoscopically trained personnel or limited access to specialist equipment.

There is increasing evidence to support the higher success rates of endoscopic-assisted probing, especially in lowering the formation of false passages [3,4].

Conventional S&P is a blind procedure and is reported to have variable success rates [7]. The creation of a false passage, unexplained failure, and traumatic stenosis or adhesions are known complications of unguided probing. Sener and Onerci suggested nasal endoscopy for proximal obstructions, which have a higher risk of false passage formation [8]. Han et al. illustrated various abnormalities at the distal end of the NLD - such as stenotic valves, thick membranes with a resultant false passage, stretchable “elastic” valves, and re-closure by redundant, ballooned nasal mucosa - as reasons for probing failure [9]. Sun et al. described Hasner valvulotomy to optimise the distal end of the NLD [10]. In a systematic review by Trott et al., conventional probing had a success rate of 75.3% in 293 eyes, whereas endoscopic-guided probing was successful in 95.3% of 162 eyes [7].

Our case series has demonstrated excellent outcomes in cNLDO with primary nasal endoscopic S&P, comparable with those of other studies [6]. The availability of an ENT surgeon in the operating theatre expands the range of treatment options available at the outset, especially in cases where probing is challenging. There is evidence to support better outcomes with intubation as a secondary intervention after failed probing (versus repeated probing), and a more dynamic management algorithm is recommended based on individual patient anatomy [11]. With over 73% of cases requiring only a single intervention, and, in effect, minimising exposure to general anaesthesia, there is a strong argument to support nasal endoscopic-guided primary S&P.

CF syndromes and Down syndrome

**Figure 1:**
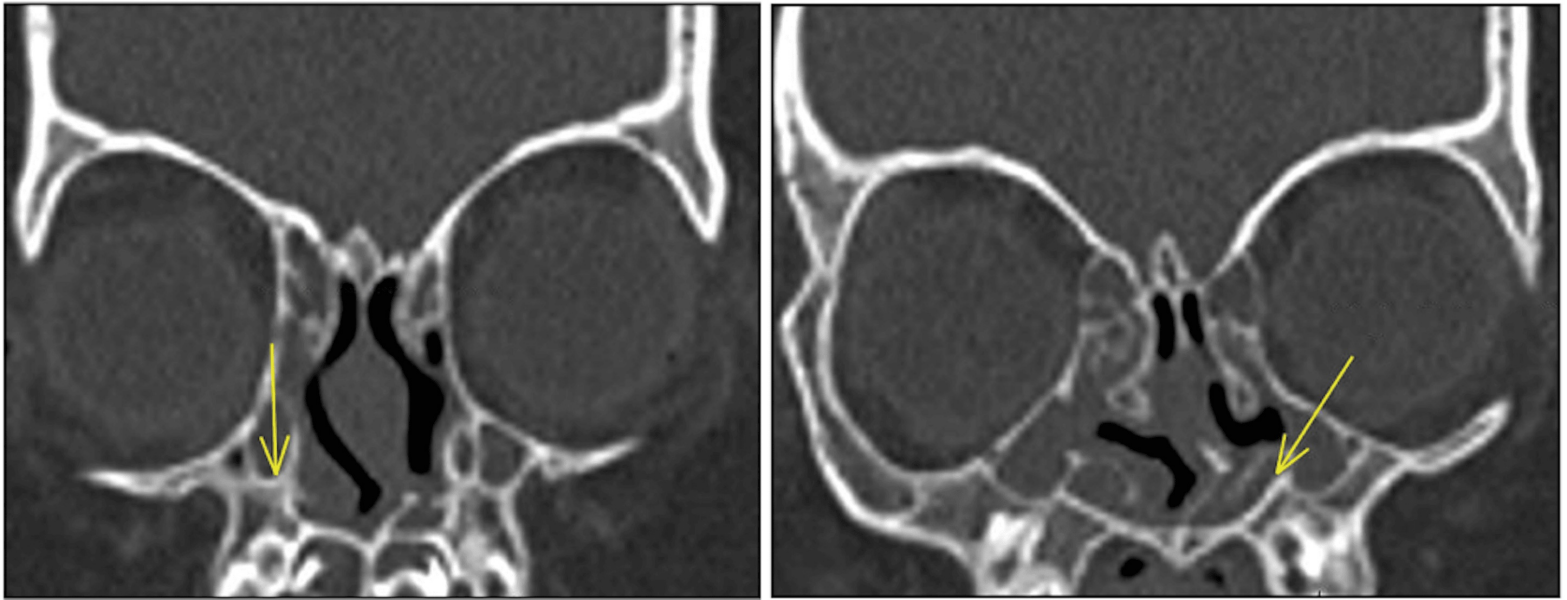
CT scan of the sinuses demonstrating complete bony stenosis at the distal ends of the bilateral nasolacrimal ducts in a patient with Saethre-Chotzen syndrome (arrows) CT: computed tomography

**Figure 2:**
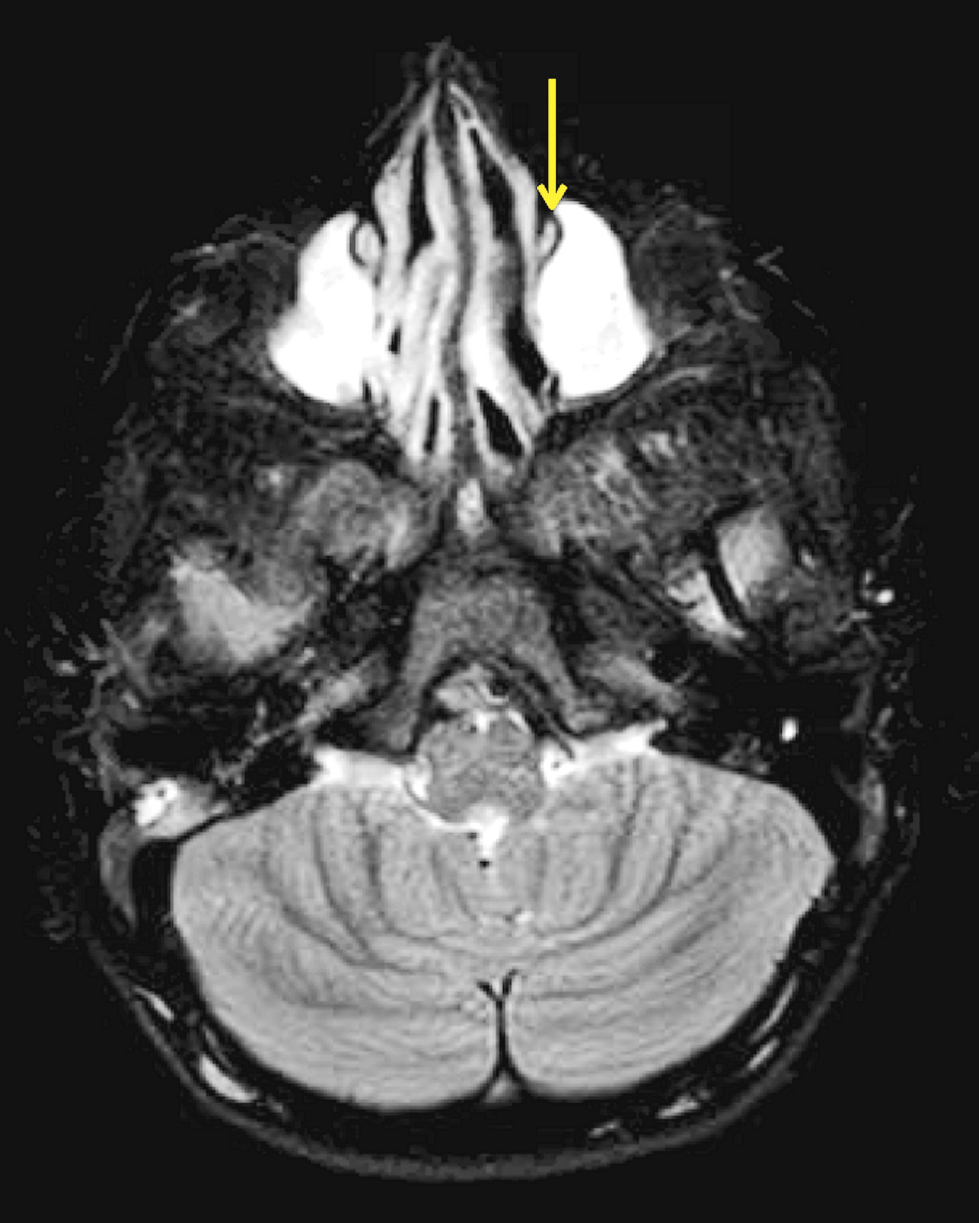
MRI STIR sequence demonstrating narrowing of nasolacrimal duct on the left side in a case with Rothmund-Thompson syndrome (arrow) MRI STIR: magnetic resonance imaging - short tau inversion recovery

We assessed the outcomes for Down syndrome and other CF syndromes separately due to the difference in the pathogenesis of NLDO in these conditions. Down syndrome is commonly associated with pre-saccal abnormalities, such as canalicular stenosis and atresia [12], whereas bony abnormalities, such as narrowing of the NLD or stenosis, are frequently encountered in CF syndromes (Figures 1-2).

We identified a statistically significant difference in the requirement for DCR between the CF and non-CF groups. The success rate of DCR in the CF group was 25%, which is comparable to the findings of Jones et al. (2007), who reported success in only 1 of 11 eyes (9%) in CF patients, highlighting the challenges of managing NLDO in this population [13]. In contrast, another study evaluating 20 DCR procedures in patients with syndromic anomalies reported a success rate of 95% [14]. However, the inclusion of a high proportion (42%) of patients with Down syndrome in that cohort may account for the more favourable outcomes.

Within our cohort of patients with Down syndrome, we observed high rates of bilateral intubation (41%) and comparatively lower success rates (50%), although interpretation is limited by the small sample size. Similar trends were reported by Landau Prat et al., who analysed 128 cases of Down syndrome and found high rates of bilateral involvement along with poorer surgical outcomes [15]. In their study, intubation and DCR were performed in 68% and 24% of patients, respectively, compared to an intubation rate of 71% in our Down syndrome cohort, with two patients currently awaiting DCR.

Given the rarity of these conditions, further prospective, multicentre studies with larger sample sizes are warranted to generate more robust evidence regarding the outcomes of NLDO management in patients with CF syndromes and Down syndrome.

Limitations

As a tertiary referral centre with a CF unit, our patient cohort may not be generalizable and is likely subject to sampling bias. Our mean follow-up period was 22.5 months, and further review will follow to assess long-term results. We had a 10% loss to follow-up rate, primarily owing to the COVID-19 pandemic, during which patients opted for patient-initiated follow-up.

## Conclusions

A multidisciplinary approach to paediatric NLDO, through combined skills and collaborative teamwork, ensures the efficient delivery of care by minimising the number of procedures and hospital attendances while optimising clinical outcomes. cNLDO can be successfully managed in the vast majority of cases with S&P, with or without intubation. The overall success rate for interventions was 87%, with a median of one procedure per case of epiphora. We recommend a low threshold for the consideration of intubation in symptomatic patients with Down syndrome and for DCR in patients with CF anomalies associated with bony anatomical abnormalities.
